# Prescription Patterns of General Practitioners in Peshawar, Pakistan

**DOI:** 10.12669/pjms.303.4931

**Published:** 2014

**Authors:** Usman Ahmad Raza, Tayyeba Khursheed, Muhammad Irfan, Maryam Abbas, Uma Maheswari Irfan

**Affiliations:** 1 Dr. Usman Ahmad Raza, MS, Assistant Professor in Community Health Sciences, Peshawar Medical College, Peshawar, Pakistan.; 2 Dr. Tayyeba Khursheed, MBBS, House Physician, Department of Medicine, Islam Teaching Hospital, Sialkot, Pakistan.; 3 Dr. Muhammad Irfan, MCPS, FCPS, MS, Assistant Professor, Dept. of Psychiatry and Behavioural Sciences, Peshawar Medical College, Peshawar, Pakistan.; 4 Dr. Maryam Abbas, MBBS, House Physician, Department of Medicine, Mufti Mehmood Teaching Hospital, D I Khan, Pakistan.; 5 Dr. Uma Maheswari Irfan, PhD, Professor of Epidemiology, College of Applied Medical Sciences, Qassim University, Buraydah, Saudi Arabia.

**Keywords:** General practitioner, Prescription, Polypharmacy

## Abstract

***Objectives:*** To find out prescription patterns of general practitioners in Peshawar.

***Methods:*** Cross-sectional survey of drug prescriptions was done at six major hospitals and pharmacies of Peshawar between April and May 2011. A total of 1097 prescriptions that included 3640 drugs, were analyzed to assess completeness, average number of drugs, prescription frequency of various drug classes, and number of brands prescribed.

***Results:*** No prescription contained all essential components of a prescription. Legibility was poor in 58.5% prescriptions. Physician’s name and registration number were not mentioned in 89% and 98.2% prescriptions respectively. Over 78% prescriptions did not have diagnosis or indication mentioned. Dosage, duration of use, signature of physician and directions for taking drugs were not written in 63.8%, 55.4%, 18.5% and 10.9% of prescriptions respectively. On average each prescription included 3.32 drugs. Most frequently prescribed drug classes included analgesics (61.7%), anti-infective agents (57.2%), multi-vitamins (37.8%) and gastrointestinal drugs (34.4%). We found 206, 130, 105 and 101 different brands of anti-infective agents, gastrointestinal drugs, analgesics and multivitamins being prescribed.

***Conclusion:*** We observed a high number of average drugs per prescription mostly using brand names, and over-prescription of analgesics, antimicrobials, multivitamins and anti-ulcer drugs. Quality of written prescriptions was poor in terms of completeness.

## INTRODUCTION

Any drug prescription should contain, in legible form, elements required for appropriate dispensing of drugs, to ensure continuity of care and for legal purposes. Rational prescription means that patients receive appropriate medicine in proper dosage, at the lowest cost.^1^ Inappropriate prescription practices like polypharmacy^2^, use of non-essential drugs^3^, indiscriminate use of analgesics, antibiotics, and vitamins^2^, ignoring important interactions, incomplete prescriptions^4^ and poor legibility^5^, are contributing to increasing antibiotic resistance^6^, adverse drug reactions^7^, serious medication errors^8^, loss of patient confidence^1^ and high cost of treatment.^1^

Polypharmacy and over-prescription of antimicrobials, analgesics, and vitamins are common in South Asia.^2^ Studies on prescription behavior in Pakistan have focused on diseases or treatment guidelines.^9^^,^^10^ This study attempts to describe the quality and patterns of drug prescriptions by general practitioners of Peshawar in terms of completeness, drugs per prescription, frequency of generic name use, and the proportion and variety of prescribed drug classes.

## METHODS

This cross-sectional study was conducted in Peshawar, Khyber Pakhtunkhwa, having a population of over three million. Prescriptions generated at six major locations in the city over a period of one month (April to May 2011) were assessed.

Location A, B and C are large public sector hospitals serving mostly middle and lower socioeconomic class. Location A receives 2700 to 3000 outpatients, Location B receives 1600 outpatients and Location C receives 1200-1500 outpatients daily (personal communication, Muhammad Irfan, 5^th^ March 2011).

Location D harbors numerous private physician clinics. These clinics receive patients from all socioeconomic classes and charge high fees.

Location E and Location F are both private non-profit hospitals located in central Peshawar. Each of these receives over 250 outpatients daily (personal communication, Usman Raza, April 2011).

Ethical clearance was given by the Institutional Ethical Committee of Peshawar Medical College. Data collection was done by a team of five medical students from clinical years under faculty supervision. Prescriptions were obtained for viewing, from patients purchasing drugs at pharmacies of the five hospitals, after obtaining informed consent. For private clinics (Location D), 25 pharmacies serving these clinics were approached.

A structured proforma was filled by observing the prescriptions and names of drugs in each prescription. Completeness was assessed using common parameters including prescriber’s identification and signature; patient’s name; date; drug name, strength, dose, form, frequency/duration of use, directions for taking the drug; diagnosis/indication for prescribed drugs. Legibility of prescriptions was also recorded.

For this study, a General Practitioner was defined as allopathic practitioner holding MBBS degree, practicing as a medical specialist or a general physician. Prescriptions of other specialists, and those for in-patients and emergency cases were excluded. Poor legibility was defined as difficulty in reading names of one or more drugs in a prescription by the data collection team in the first attempt.^11^

Data were entered into an online database (based on MySQL) and double checked. Database was imported into Microsoft Excel 2010 for analysis. Averages or proportions were calculated for variables and presented as graphs or tables. Of the 1103 prescriptions, eight were discarded due to incompleteness, yielding a final sample of 1097 prescriptions.

## RESULTS

A total of 1097 prescriptions written by general practitioners that included 3640 drugs were analyzed. Only 373 (10.25%) drugs were prescribed using generic names. The average number of drugs per prescription was 3.32 ± 1.2 (Table-I). The maximum number of drugs recorded in a prescription was 11. More than 70% prescriptions contained three or more drugs.

**Table-I:** Drugs per prescription

*Location*	*Type of facility*	*Prescriptions*	*Average (median)drugs/prescription*	*Maximum drugsin a prescription*
A	Public	89	2.76 (3)	6
B	Public	224	3.31 (3)	10
C	Public	462	3.15 (3)	7
D	Private for-profit	123	4.07 (4)	11
E	Private non-profit	88	3.14 (3)	8
F	Private non-profit	111	3.82 (4)	7
All	-	1097	3.32 (3)	11

Elements considered essential for the medical and legal completeness and usefulness of a written prescription^4^were assessed in our study, as depicted in Table-II.

**Table-II:** Quality of written prescriptions (n = 1087)*

*Parameter of Quality*	*Frequency (%)*	*Range* **
Physician’s name not written	967 (89.0%)	32.1 – 99.1%
Signature of physician absent	201 (18.5%)	3.5 – 47.4%
Physician’s Registration No. not written	1067 (98.2%)	96.4 – 100%
Patient's Name not mentioned	27 (2.5%)	0 – 8.9%
Date of prescription not written	75 (6.9%)	0.5 – 15.4%
Poor legibility	636 (58.5%)	17.7 – 67.9%
Diagnosis not written	856 (78.7%)	8.0 – 89.7%
Dosage not written	694 (63.8%)	44.6 – 69.2%
Dosage form not mentioned	95 (8.7%)	0 – 24.4%
Duration of use not written	602 (55.4%)	24.8 – 73.4%
Directions for taking drugs not written	119 (10.9%)	0.9 – 19.2%

* For these results, 10 incomplete records were not considered yielding a sample size of 1087

** Range refers to highest and lowest values among the six locations sampled

**Fig.1:**
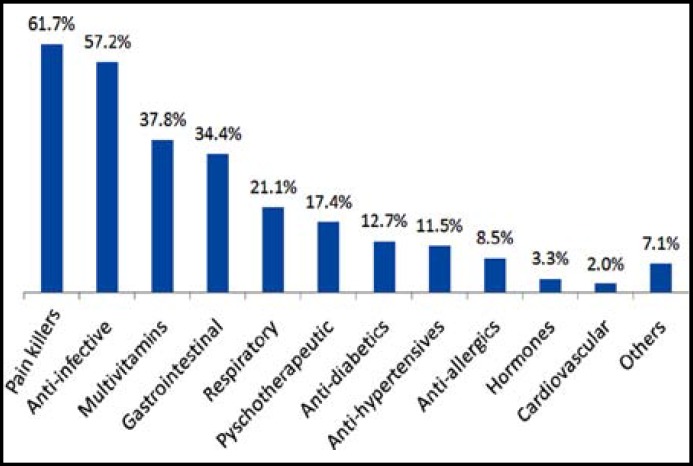
Percent prescriptions containing various drug classes

As depicted in Fig.1, analgesic drugs were most frequently prescribed (61.7%), followed by anti-infective agents (57.2%), vitamin supplements (37.8%) and gastrointestinal drugs (34.4%). The ‘Others’ category included vaccines, electrolyte fluids, immune-modulators, local anesthetics, dermatological creams etc.

Among individual drugs, most commonly prescribed were various brands of Multivitamins (304, 8.4%), Paracetamol (250, 6.9%), Diclofenac (212, 5.8%), Omeprazole (116, 3.2%), Levofloxacin (115, 3.2%) and Ibuprofen (102, 2.8%). A large variety of brands was found among prescriptions especially for anti-infective agents, gastrointestinal drugs, analgesics, multivitamins and psychotherapeutics with 206, 130, 105, 101 and 71 different brands being prescribed in these classes respectively.

## DISCUSSION

The World Health Organization has recommended an ideal average upper limit of 2.0 drugs per prescription.^12^ Our result of 3.32 drugs per prescription suggests prevalent polypharmacy. Other countries show similar figures ranging from 2.2 to 4.34.^13^^-^^15^ Bangladesh and Yemen however, report figures of 1.44 and 1.5 respectively^16^^,^^17^ which has been attributed to successful implementation of a well-defined drug policy.^16^ Polypharmacy is known to cause unnecessary adverse reactions, drug interactions, and complications.^8^

The overall quality of written prescriptions was poor, since no prescription contained all essential components of prescription. Similar to our results, most prescriptions did not include the physician’s name, signatures or registration numbers in studies from Nepal and India.^18^^,^^19^ The absence of these details in prescriptions renders them legally questionable.

Poor hand writing of practitioners can lead to fatal instances of inadvertent drug substitutions.^8^ Less than half the prescriptions in our study showed good legibility, which is comparable to a study from United Arab Emirates^15^, but worse than other studies in the region.^19^^,^^20^ Marked variation in legibility was observed among the locations we sampled. Interestingly, the prescription format for one location (with legibility above 82%) was found to be well structured as compared to that in other locations.

The absence of a diagnosis or indication makes it difficult to assess rationality of a prescription, and may lead to repeat testing and treatment, increasing financial burden on the patient. Only a fifth of prescriptions in our study contained a diagnosis or indication, which is lower than figures reported from India.^19^

About two third of prescriptions in our study did not specify the dose of drug, which is higher than a reported figure of 19% from Nepal.^18^ Over half of prescriptions in our study did not mention dosage duration, which is lower than 69% reported from India.^19^ Almost 11% of our prescriptions lacked directions for taking drugs, which is close to that in Nepal^18^ and much better than the 88% reported from India.^19^

Inconsiderate antimicrobial prescription may contribute to the emergence of antimicrobial resistance. Compared to our figure of 57.2%, studies from India quote antibiotic prescription frequencies as low as 9.6%^21^ to as high as 43%.^17^^,^^22^ Varying figures have been reported in other countries such as 25% in Bangladesh^16^, 31% in the United Arab Emirates^23^, 39% in China^24^, 56% in Uganda^17^, 63% in Sudan^17^ and 72% in Nepal.^13^ Comparable percentage of 54% to 62% has been reported in another study from Pakistan.^14^ Considering that 90% of these prescriptions did not contain any indication, it is difficult to rationalize this high frequency of antimicrobials prescription, and concerns arise about their appropriateness. Frequency of analgesic prescription in our study is the highest reported among studies from other countries (11.9% to 30%)^13^^,^^19^^,^^22^^,^^23^^,^^25^ for analgesics prescription. Compared to multivitamin prescription of our study (37.8%) other studies in the region report varying frequencies from 5.9% to 50%.^2^^,^^15^^,^^19^^,^^21^^,^^22^^,^^25^ The high prescription frequency of antibiotics, analgesics, GI drugs, multivitamins and psychotherapeutics may indicate tendency of physicians towards symptomatic relief rather than curative treatment.

Use of generic drug names is recommended worldwide, but was very low in our study. Disappointing figures were found in most studies ranging from 2% to 43.9%^2^^,^^4^^,^^15^^,^^19^^,^^23^^,^^25^ with exceptions of China^24^ and Bangladesh^16^ reporting 69.2% and 78% respectively. We observed very large number of brands of various drug classes being prescribed, which may be linked to the competitive market of pharmaceuticals and weak regulatory systems, which have not been able to cap the number of brands being produced.

The practices reported in our study may lead to higher costs, poor quality of care, emerging antimicrobial resistance and unnecessary health risks due to adverse reactions and drug interactions.

Our study excludes specialist consultants, non-registered practitioners and in-patients, and the results represent a part of the full spectrum of drug prescriptions in the region. Further, owing to the limited data collection period, it does not account for seasonal variations.

## CONCLUSION

Our study found a relatively high number of drugs per prescription, with a high proportion of brand name prescriptions. We found high frequencies of analgesic, antimicrobial, multivitamin and gastrointestinal drug prescription. In terms of quality, none contained all essential components of a prescription. Legibility was poor and essential elements missing in many prescriptions. Results indicate the need to study factors associated with these practices and promote evidence-based prescription.

## Notes

***Source of funding:*** Departmental activity.
